# Determining Optimal Fractionation of Neoadjuvant Radiation in Low-Risk, Early-Stage Breast Cancer—Randomized SIGNAL Clinical Trial

**DOI:** 10.3390/cancers18121867

**Published:** 2026-06-08

**Authors:** Melanie Spears, Michael Lock, Brian Yaremko, Vida Talebian, Zoe Kerhoulas, Kalan S. Lynn, William T. Tran, Neil Gelman, Matthew Mouawad, Stewart Gaede, Allison Maciver, Megan Hopkins, Linda Liao, Fang-I Lu, Anat Kornecki, Silvia C. Formenti, Sandra Demaria, Muriel Brackstone

**Affiliations:** 1 Ontario Institute for Cancer Research, MaRS Centre, Toronto, ON M5G 1M1, Canada; mspears@oicr.on.ca (M.S.);; 2 Department of Laboratory Medicine and Pathology, University of Toronto, Toronto, ON M5S 1A8, Canada; 3 Lawson Health Sciences Research Institute, London, ON N6A 5W9, Canada; 4 Division of Radiation Oncology, Western University, London, ON N6A 5W9, Canada; 5 Medical Biophysics, Western University, London, ON N6A 5C1, Canada; 6 St. Joseph’s Health Care Centre, London, ON N6A 4V2, Canada; 7 Department of Radiation Oncology, University of Toronto, Toronto, ON M5T 1P5, Canada; 8 Sunnybrook Research Institute, Toronto, ON M4N 3M5, Canada; 9 Department of Medical Imaging, Western University, London, ON N6A 5C1, Canada; 10 Department of Surgery, Western University, London, ON N6A 5C1, Canada; 11 Department of Radiation Oncology, Weill Cornell Medicine, New York City, NY 10065, USA

**Keywords:** tumor-infiltrating lymphocyte, immune-oncology, breast cancer, stereotactic body radiotherapy, gene expression, clinical trial

## Abstract

Background: Neoadjuvant partial breast irradiation using stereotactic body radiotherapy (SBRT) has emerged as a strategy to induce tumor and immune responses in early-stage, low-risk breast cancer. While prior studies have demonstrated encouraging response rates and evidence of immune modulation, the optimal radiotherapy regimen for immune priming remains unclear. SIGNAL 2.0 is a randomized phase II trial designed to compare the biological and immunological impact of a single-fraction versus three-fraction neoadjuvant SBRT. Materials and Methods: Sixty-one postmenopausal patients ≥ 50 years with unifocal, hormone positive, node-negative invasive ductal carcinoma < 3 cm were randomized 1:1 to receive either 21 Gy in one fraction or 30 Gy in three fractions, delivered to the tumor in the prone position. Core biopsies were collected pre-SBRT and 14–20 days post-SBRT at the time of surgery. Immune markers were assessed using tumor-infiltrating lymphocyte (TIL) scoring, NanoString nCounter PanCancer Immune Profiling, and NanoString GeoMx Digital Spatial Profiling (DSP). Results: Available tumor samples from 47 patients underwent paired tissue analysis. Three-fraction SBRT induced 200 differentially expressed genes, including enrichment of pathways related to adaptive immune activation, with significant increases in expression levels of macrophages, dendritic cells, neutrophils and CD8 T-cells. Proteomic profiling also identified a significant increase in the expression levels of neutrophils, Treg cells, macrophages, and NK cells in the tumor microenvironment of the samples from patients receiving the three-fraction regimen. Conclusions: Neoadjuvant SBRT induces measurable immune activation, with three-fraction regimens generating more extensive transcriptional, proteomic, and cellular immune changes than a single fraction. Three-fraction neoadjuvant SBRT may provide superior immune priming, providing a foundation for future trials integrating neoadjuvant radiotherapy with immunomodulatory therapies.

## Simple Summary

Radiation delivered prior to surgery in patients with early-stage, low-risk invasive breast cancer has been used to reduce local recurrence. While research is ongoing to evaluate whether it can also prime the immune system, data was lacking on the optimal radiation dosing required to upregulate the immune system within the tumor. We randomized 61 eligible patients to 21 Gy in one fraction versus 30 Gy in three-fractions and all patients tolerated either treatment without any significant side effects. Forty-seven patients had pre- and post-radiation tumor biopsies available for analysis with bulk RNA sequencing and spatial proteomic profiling to evaluate immune cell pathway upregulation. Patient tumors treated with three fractions of radiation demonstrated significant upregulation in gene expression and proteomic pathways for macrophages, Treg cells, NK cells, and dendritic cells. Future clinical trials using preoperative radiation for immune priming should consider a regimen of three fractions, such as 10 Gy per fraction, to the tumor.

## 1. Introduction

Standard treatment for early-stage hormone receptor positive breast cancer (HR+ BC) typically involves breast conserving surgery (BCS) followed by whole-breast radiotherapy (WBRT), or accelerated partial breast radiotherapy, or stereotactic body radiotherapy (SBRT) [1] along with adjuvant endocrine therapy and/or chemotherapy, depending on tumor phenotype and risk of recurrence. The advantages of SBRT include delivering high doses of radiation over a small number of fractions, reducing toxicity to healthy breast tissue, and lower overall treatment cost due to a reduced number of visits.

Ultrahypofractionated SBRT was popularized following several trials implementing intraoperative radiotherapy (IORT). The TARGIT trial [2] evaluated the use of a single fraction of IORT at the time of surgical lumpectomy where the tumor bed received 20 Gy. It demonstrated that IORT was non-inferior to standard external beam radiotherapy (EBRT) with no statistically significant differences in recurrence-free survival, distant disease-free survival and overall survival. The ELIOT trial [3] was a similar study designed to deliver 21 Gy fraction of IORT with electrons and demonstrated similar initial response, although longer-term follow-up demonstrated a significant increase in local recurrence in the IORT group (11% versus 2% in WBRT group), albeit without any difference in overall survival [4]. The use of IORT requires specialized equipment not available in many hospitals and therefore would be cost prohibitive for many health care systems. Further studies then demonstrated acceptable rates of local control and toxicity with single fraction preoperative EBRT to the tumor [5], including our pilot study where we treated low-risk, early-stage invasive breast cancer in the prone position with 21 Gy in one fraction [6]. This single fraction was well tolerated without any significant skin or other toxicity and provided excellent cosmetic results and patient satisfaction. At the same time, proof-of-principle studies demonstrated that using radiotherapy to prime T cell responses against tumor antigens could be translated to clinical care [7], creating an interest in using pre-operative radiotherapy to induce immune priming [8] with the hopes of inducing an immune memory that may reduce the risk of cancer recurrence.

Historically, radiotherapy has been used in cancer with the purpose of directly killing the cancer cells. More recently, there is growing data to support that radiation is triggering the release of both pro-inflammatory (and anti-inflammatory) mediators, by both increasing tumor infiltrating immunostimulatory (and immunoinhibitory) cells and enhancing the expression of neoantigens [8]. Localized radiotherapy may also promote dendritic cell maturation and activation and enhance phagocytosis of cells by antigen-presenting cells’ (APCs) increased presentation of tumor-associated antigenic peptides and T cells being primed from naïve towards memory phenotypes [8]. Breast cancer cells, however, have traditionally been considered immunologically inactive due to a combination of factors including but not limited to low mutational burden [9], limited T cell infiltration and immunosuppressive cytokines controlling the tumor microenvironment (TME) [10]. Studies of the breast cancer TME suggest that some tumor phenotypes (such as HR+) may be more immunologically “cold” compared to the triple-negative and HER2-positive subtypes [11,12], based on response to checkpoint inhibitor immunotherapy using anti-programmed cell death protein 1 (PD-1) monoclonal antibody therapies, including in the neoadjuvant setting [13].

Recent research has been investigating the role of radiotherapy in potentiating the response of cancers to immunotherapy. This was first demonstrated in mouse models, demonstrating improved response to anti-CTLA4 with the addition of radiotherapy [14]. Understanding the role of radiotherapy in inducing an abscopal response [15] has led to a number of studies designed to increase our understanding of the role of tumor-infiltrating lymphocytes in immunogenic responses to treatment [16,17,18,19]. While the initial focus was on tumors expressing high levels of PD-1, Bruss et al. demonstrated that neoadjuvant radiotherapy delivered to humanized tumor mice (HTM) enhances anti-PD-L1 efficacy across breast cancer subtypes [20], thus providing an opportunity to exploit radiotherapy for the purposes of turning a ‘cold’ tumor into a ‘hot’ one.

What remains unknown is the dose and fractionation of radiotherapy required to optimally induce immune priming when delivered in the preoperative setting for breast cancers. Data from animal studies had suggested that three fractions of 8 Gy per fraction were optimal for immune priming in breast cancer [21] and there have been clinical trials utilizing three-fraction regimens in the neoadjuvant setting [22] but there are no known trials confirming which fractionation is optimally immune priming in humans. The purpose of this study was to compare single versus fractionated radiation and explore which radiotherapy dosage schedule provided a superior immune priming response in early-stage breast cancers [23,24]. We selected early-stage, hormone receptor (HR) positive cancers as they are most commonly treated with upfront surgery. Additionally, this tumor phenotype has been traditionally considered immune ‘cold’, and we wanted to evaluate whether preoperative radiotherapy could induce immunogenicity in this tumor subtype, which could then expand treatment options for the most common breast cancer phenotype to include immunotherapy. In this single site randomized phase II trial, patients with early-stage HR+ breast cancers were treated with either one or three fractions of neoadjuvant SBRT to the tumor followed by lumpectomy, using dosimetry constraints outlined in our prior SIGNAL 1.0 pilot trial [6]. Tumor samples taken before and after SBRT (prior to surgery) were analyzed for immune response as measured by gene expression and digital spatial profiling of the tumor microenvironment.

## 2. Materials and Methods

### 2.1. Clinical Trial Conduction

The SIGNAL 2.0 breast cancer trial was approved by the University of Western Ontario’s Health Sciences Research Ethics Board (HSREB #105643) and made available on www.clinicaltrials.gov (NCT02212860). This randomized phase II clinical trial was conducted in London, Canada, with enrollment of patients taking place at St. Joseph’s Health Care London and SBRT taking place at the London Regional Cancer Program. Enrollment occurred between October 2017 and March 2020. Patients presenting with a new diagnosis of invasive breast cancer were deemed eligible if they were age 50 or older and postmenopausal, had a unifocal tumor less than 3 cm in size on pre-treatment imaging which was ER positive, Her2 negative and clinically node negative. Tumors were required to be in a location where a minimum 2 mm surgical margin could be obtained, were able to lie in the prone position with arms raised above their heads for the duration of the treatment period and were able and willing to have surgery within 14–20 days of SBRT. Patients were deemed ineligible if they did not meet eligibility criteria, had previous RT to the same breast, had suspicious diffuse calcifications in the target breast, were suspected of having distant metastatic disease or lymph node involvement, skin involvement, Paget’s disease of the nipple, not able to receive RT or not appropriate for breast conserving surgery or if they had any other malignancy in the last five years.

The dosage of 21 Gy in one fraction was based on our prior published work demonstrating a biologically equivalent dose (BED) to the then-standard 50 Gy in 25 fraction whole breast radiotherapy, demonstrating equivalent tumor control and acceptable toxicity using α/β of 10 Gy for calculations of equivalent early effects and an α/β of 3 Gy for calculations of equivalent late effects. The dosage of 30 Gy in three fractions has a biologically equivalent dose to the standard 50 Gy in 25 fractions. Radiotherapy was prescribed as follows, using contouring guidelines in accordance with standard international recommendations and the Radiation Therapy Oncology Group (RTOG)-endorsed consensus for delineation [25]. Planned tumor volume (PTV) represented clinical tumor volume (CTV) plus 5 mm margin. In dosimetry planning, 95% of PTV was to be covered by the 100% isodose line in the 21 Gy in 1 fraction arm or by the 100% isodose line of 10 Gy in each of the three-fractions in the 30 Gy arm.

**Figure 1:**
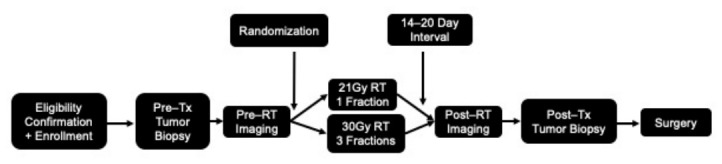
SIGNAL 2.0 Study Schema. RT = radiotherapy; Tx = treatment.

The study schema is outlined in Figure 1. Eligible patients underwent a CT Simulation in the prone position and were excluded from study participation if dosimetry constraints could not be met. Breast images from 3T PET/MRI were used to confirm complete tumor coverage with contouring. Eligible patients were then randomized 1:1 by a research coordinator using a random number generator, with one cohort being treated with one fraction of SBRT (21 Gy in one fraction) to the breast tumor PTV and the other cohort being treated with three fractions (30 Gy in three fractions). Patients were asked to provide a core tissue sample prior to radiotherapy. After randomization, patients were assigned to their treatment arm and were simulated and treated prone, to receive either one or three fractions of SBRT. Fourteen to 20 days after the last fraction of radiotherapy was delivered, the participants underwent an image-guided post-treatment core biopsy procedure. Shortly thereafter, all patients received a breast conserving lumpectomy ± sentinel lymph node biopsy as per clinical standard. A total of 80 participants were enrolled and 61 met dosimetry constraints and were successfully treated (one bilaterally). Post-operative follow-up was completed three to four weeks after surgery, six months after surgery, and 12 months after surgery (with mammogram at one year), during which participants were evaluated for any treatment toxicity. Any events (recurrence or death) were captured at 5 years. Of the 61 patients treated in this study, pre- and post-treatment tumor tissue was available in 47 patients. The remaining patients had their pre-treatment core biopsy at another institution which was therefore not available for analysis. Fresh frozen and paraffin embedded (FFPE) slides were made and hematoxylin and Eosin staining was used to identify cancer cells.

### 2.2. Tumor Infiltrating Lymphocyte (TIL) Evaluation

Stromal tumor-infiltrated lymphocytes (sTILs) were scored by one trained pathologist on H&E-stained slides according to the guidelines of the International Immuno-Oncology Biomarker Working Group [26]. Paired pre-treatment and post-treatment tumor tissue was available for 38 patients, 20 treated with 21 Gy in one fraction and 18 treated with 30 Gy in three fractions.

### 2.3. RNA Isolation and Gene Expression Profiling

To profile the changes in immune response to radiation, we performed NanoString gene expression profiling. We successfully analyzed 24 pairs of frozen tumors in the 21 Gy in one fraction arm and 23 pairs of frozen tumors in the 30 Gy in three fraction arm. Total RNA was extracted from the frozen tissue using RNeasy Kit (Qiagen Inc., Toronto ON, Canada), per manufacturer’s instructions. RNA profiling was performed with 200 ng (quantified using NanoDrop-1000, Thermo Fisher Scientific, Waltham, MA, USA) of RNA using the NanoString nCounter Human V.1.1 PanCancer Immune Profiling Panel (NanoString Technologies Inc., Seattle, WA, USA), according to the manufacturer’s instructions.

### 2.4. Immune Infiltration Signature Analysis

Gene annotation data for the nCounter PanCancer Immune Profiling Panel was obtained from the NanoString product site. This panel encompasses gene signatures for immune cells such as B cells, T cells, CD8 T cells, CD45, cytotoxic cells, dendritic cells, exhausted, macrophages, mast cells, neutrophils, NK CD56dim cells, NK cells, Th1 cells, Treg. For each cell, a set of genes was assumed to be specific to its cell type. These genes and corresponding cell types are listed in Supplementary Table S1. Immune profiles were subsequently generated from normalized mRNA gene expression levels [27].

### 2.5. Digital Spatial Profiling

Whole slides were prepared following the GeoMx DSP Slide Preparation User Manual v2.1 (NanoString Technologies, Inc.) [27]. Immunostaining was performed using a cocktail of antibodies comprising an 18-plex core panel for immune cell profiling, supplemented by four additional modules: immuno-oncology drug targets (10-plex), immune activation status (8-plex), immune cell typing (7-plex), and pan-tumor (9-plex). Morphological markers included anti-PanCk and anti-CD45, and nuclear counterstaining was performed with SYTO 13 (GeoMx Nuclear Stain Morphology Kit, NanoString; full antibody list in Supplementary Table S2). Stained slides were processed on the GeoMx DSP instrument. Circular regions of interest (ROIs) of 690 µm in diameter were defined, and the visualization markers PanCk and CD45 were used to segment each ROI into tumor and tumor microenvironment (TME) compartments, respectively.

### 2.6. nCounter Hybridization Assay for Photocleaved Oligo Counting

Four quality control parameters were used and standardized across all experiments to minimize technical variability. Field of View (FOV) registration was set to capture at least 75% of FOVs during barcode counting by the digital analyzer. Binding density, defined as the number of barcodes per micron, was used as a quality check, with values set based on the instrument’s defined best practices. The binding density was maintained between 0.1 and 2.25 for the gene expression assay and between 0.3 and 3.0 for the protein assay, serving to normalize lane-to-lane and cartridge-to-cartridge variation arising from differences in experimental days, magnetic bead purification, and probe-slide binding. Positive control probe hybridization counts were required to reach a minimum of 100 prior to positive control normalization. Minimum thresholds of 20 nuclei and a surface area of 1600 µm^2^ were also enforced. Samples failing any of these criteria were flagged and excluded from downstream analysis.

Samples passing quality control underwent two sequential normalization steps. First, counts were scaled to the surface area of each compartment by applying the ratio of the geometric mean to the respective tumor or TME surface area. Second, counts were normalized to the housekeeping proteins S6, GAPDH, and Histone H3. Rabbit and mouse isotype controls were included as negative controls to correct for background signal.

### 2.7. Statistical Analysis

Raw gene expression data were processed using NanoString’s nSolver v4.0 software with the Advanced Analysis 2.0 plugin. Normalization was carried out using internal negative control probes, synthetic positive controls, and 36 housekeeping genes selected by the nSolver normalization module via the geNorm algorithm [28,29]. Normalized counts were log2-transformed prior to further analysis. Multiple testing correction was applied using the Benjamini–Hochberg (BH) method to control the false discovery rate (FDR). Differentially expressed genes (DEGs) were defined as those with a log2 fold-change greater than 1 or less than 1 and a BH-adjusted q-value below 0.05. GeoMx DSP Analysis Suite version 3.1.0.222 was used for quality control, normalization and analysis of the protein spatial data [30]. The housekeeping proteins S6, H3, and GAPDH were used for normalization. Normalized protein levels were used for the downstream analysis [31]. The immune markers from the target groups annotation were provided by GeoMx (Supplementary Table S2).

## 3. Results

Patients were treated in accordance with randomization assignment as outlined in the study schema (Figure 1). Table 1 illustrates the patient demographics in both treatment arms. While 61 patients were treated across both arms in the clinical trial, only the tumor samples from 47 of these patients were available for analysis as the other 14 patients received their diagnostic tumor biopsy at another institution and therefore no fresh frozen tumor was available for pre-treatment analysis. Patient demographic and tumor data are listed both for the two clinical cohorts and the subset of each cohort for which molecular analyses were conducted. There were no notable or significant differences between patients in the two treatment arms. The average age of these patients was 65, with primary T1 tumors averaging 11 mm on clinical exam and 9 mm at pathological evaluation. Most were grade 1 or 2, with one patient in each treatment arm being upstaged to grade 3 at the time of surgical pathology evaluation. Six of the 50 patients who underwent sentinel node biopsy in the clinical trial were found to be node positive at pathology (three in each arm), and four of these patients were treated with adjuvant chemotherapy (one in 21 Gy arm and three in 30 Gy arm). These four patients also received additional adjuvant whole breast radiotherapy (50 Gy in 25 fractions using standard two-field tangents). In total, 23 patients were treated with adjuvant endocrine therapy.

There were no significant treatment-related toxicities and no grade 3 or higher skin or breast toxicities. Patients tolerated the preoperative radiotherapy and breast conserving surgery. One postoperative wound infection was noted in this study. All patients self-reported high satisfaction with their cosmetic outcomes. At five years, no patients experienced any recurrences (local, regional or distant) in either treatment arm.

Baseline sTILs ranged from 0% to 50% (Table 2) with 66% of cases with 0 or 1% sTILs, consistent with the fact that HR+ BC is an immunologically cold tumor. Post-RT, an increase in sTILs was seen in 17 cases (9 in 21 Gy in one fraction and 8 in 30 Gy in three fractions), whereas 14 cases showed no change, and 3 cases showed decreased sTILs. Four cases could not be evaluated because there was no tumor left in the lumpectomy specimen. Overall, 50% of the patients with residual tumor in the lumpectomy specimen showed an increase in sTILs compared to baseline, with no difference between the two treatment arms. Where an sTIL increase was seen, it was relatively modest, going from 0 or 1% to 5 or 10% for the majority of patients.

### 3.1. mRNA Expression Profiling

**Figure 2:**
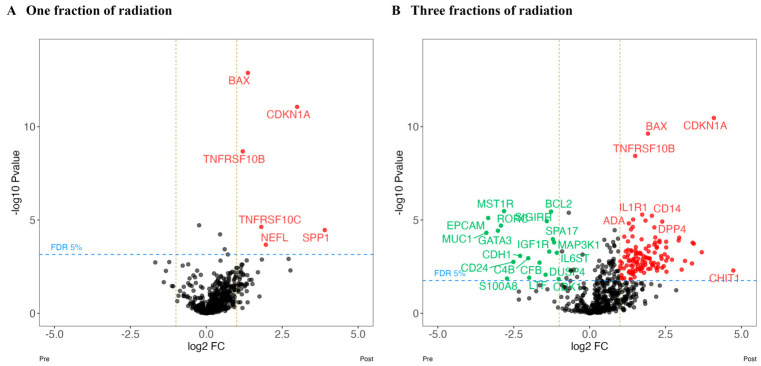
Three fractions of radiation result in markedly increased changes in gene expression. Volcano plots show differential gene expression comparing post-treatment samples versus pre-treatment. The Y-axis represents the negative logarithm base 10 of *p*-values, and the X-axis represents the log2 of fold change ratio of post treatment levels versus pre-treatment. Gene with a log2 fold change greater than 1 and lower than −1, as well as FDR < 0.05 are labeled on the figure. Red labels represent genes up-regulated after treatment, and green labels represent the down-regulated genes after treatment. (**A**) Patients treated with 21 Gy of radiation in one fraction. (**B**) Patients treated with 30 Gy of radiation in three fractions.

We observed notable differences in the immune microenvironment gene expression patterns between samples obtained from tumors pre- and post-treatment (Figure 2). We identified a total of 175 differentially expressed genes (DEGs) (173 upregulated and 2 downregulated) using a 5% adjusted *p*-value across the entire patient population regardless of treatment arm (Supplementary Table S1). In patients who received 21 Gy in one fraction, we identified 10 DEGs (9 upregulated and 1 downregulated). In comparison, patients who received 30 Gy in three fractions had 200 DEGs (173 upregulated and 27 downregulated). Genes that had the highest change in expression included SPP1, CDKNA1, LBP, MARCO, and NEFL. There were significantly more upregulated genes in the 30 Gy in three fraction arm (Figure 2).

**Figure 3:**
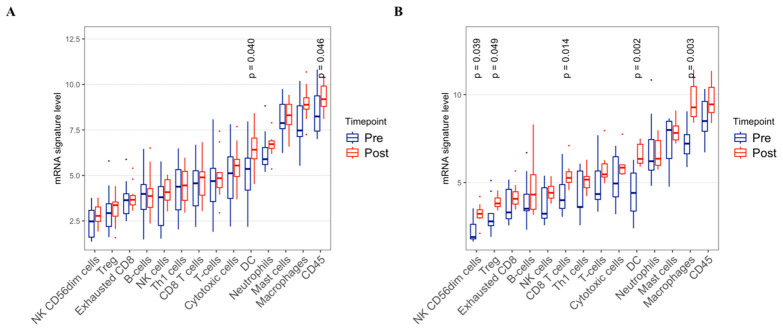
**Three fractions of radiation result in changes in the tumor microenvironment.** Box-plots showing the expression levels of immune cells present in pre-(blue) and post- (red) treated samples. (**A**) Patients treated with 21 Gy of radiation in one fraction. (**B**) Patients treated with 30 Gy of radiation in three fractions.

The immune profile based on mRNA gene expression data showed that there were significant changes in the cellular composition after radiotherapy (Figure 3). In patients who received only one fraction of radiation, there was a significant increase in dendritic cell markers (*p* < 0.05). In patients who received three fractions, there were significant increases in expression levels of macrophages (*p* < 0.01), dendritic cells (*p* < 0.01), and CD8 T cells (*p* = 0.014).

**Figure 4:**
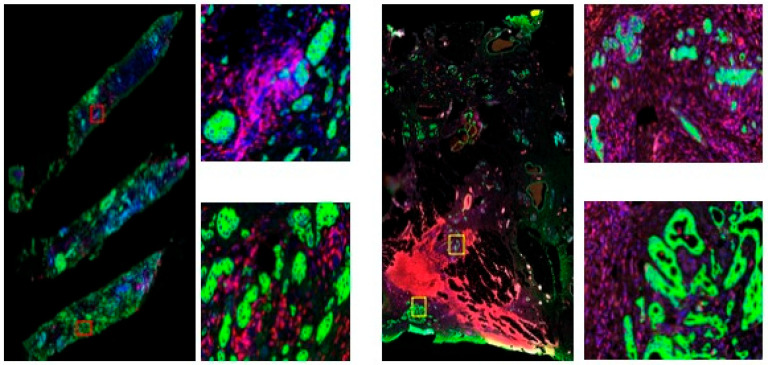
**Representative images of NanoString GeoMx DSP compartment selection in SIGNAL 2.0 cohort.** Low-resolution immunofluorescence image of the markers that define the selected compartments. Selection of tumor compartment (pan-CK, green) and TME compartment (CD45, red).

To further explore immune upregulation in the tumor versus tumor microenvironment (TME), GeoMx DSP was performed (Figure 4).

**Figure 5:**
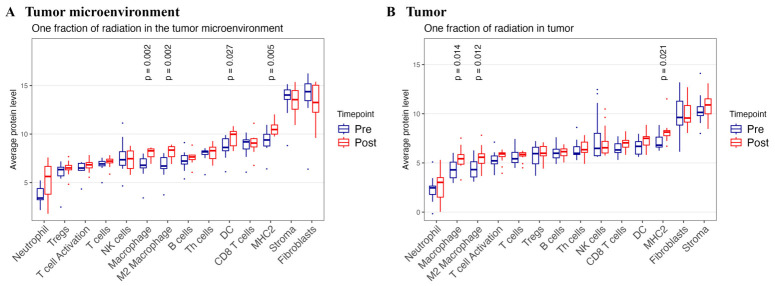
**One fraction of radiation results in modest changes in the tumor microenvironment.** Box-plots show the average protein levels that represent each immune cell type for pre-treated (blue) and post-treated (red) samples. (**A**) The results for tumor microenvironment compartment. (**B**) The results for tumor compartment.

The immune cell profiles based on protein data showed that there were significant changes in the cellular composition after radiotherapy (Figure 5). Patients treated with 21 Gy in one fraction demonstrated an increase in the expression of M2 macrophages within the tumor compartment (*p* < 0.01). Within the TME compartment, there were increased expression levels of macrophages and M2 macrophages observed (*p* < 0.01).

**Figure 6:**
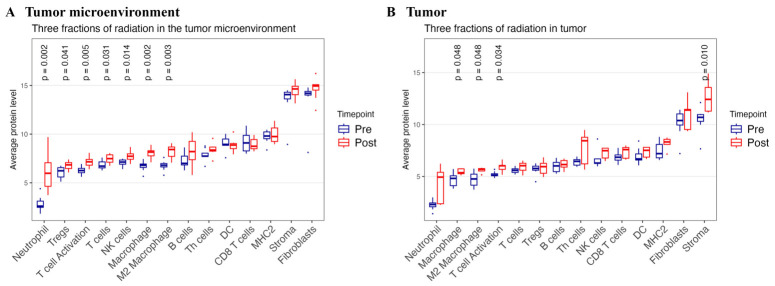
**Three fractions of radiation result in markedly larger changes in immune cell expression in the tumor microenvironment.** Box-plots show the average protein levels that represent each immune cell type for pre-treated (blue) and post-treated (red) samples. (**A**) The results for tumor microenvironment compartment. (**B**) The results for tumor compartment.

In patients treated with 30 Gy in three fractions, immune cells within the tumor compartment demonstrated a significant increase in the expression levels of neutrophils, macrophages, M2 macrophages, NK cells, and stromal cells (*p* < 0.01) (Figure 6). Within the TME compartment, there was a significant increase in the expression levels of neutrophils, Treg cells, macrophages, M2 macrophages, and NK cells (*p* < 0.01).

### 3.2. Proteomic Profiling

**Figure 7:**
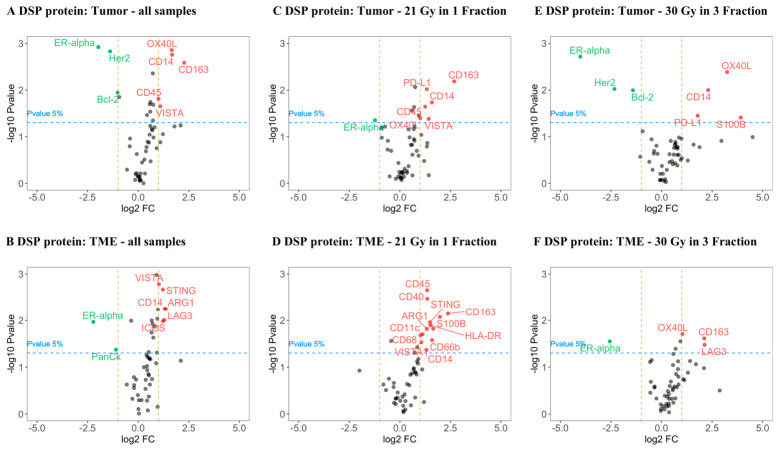
**Proteomic profiling of tumor versus tumor microenvironment (TME) by treatment arm.** Volcano plots showing protein expression in tumor and TME overall (**A**,**B**) then stratified by fractionation: 21 Gy in one fraction (**C**,**D**) and 30 Gy in three fractions (**E**,**F**). DSP = Digital spatial profiling; TME = tumor microenvironment.

In total, 61 tumor and immune proteins were profiled in the SIGNAL 2.0 cohort. Protein expression levels were successfully analyzed in 44 patients. A high correlation among the immune markers in the TME compartments was observed, indicating that multiple immune cell subpopulations were present. Within the tumor compartment there were several pan-tumor proteins that were significantly changed post radiotherapy (Figure 7A). Immune markers such as CD163, S100B, LAG3, ICOS, and CD40 exhibited the greatest increase in expression in the TME compartment (Figure 7B).

In patients treated with 21 Gy in one fraction, a downregulation of ERα was observed post radiation in the tumor compartment (Figure 7C). However, within the tumor compartment for this treatment arm, several immune markers were elevated including CD163, CD14, CD45, VISTA, PD-L1 (Figure 7D). These immune markers suggest that macrophages and T cells were infiltrating the tumor compartment. Within the TME compartment we see several immune markers elevated including CD163, ICOS, and HLA-DR. These markers indicate that more macrophages, T cells, and DC cells are present in the TME.

In patients treated with 30 Gy in three fractions, tumor markers in the TME such as ERα, HER2, and Bcl-2 are significantly elevated compared to pre-treatment samples (Figure 7E). Immune markers such as OX40L and CD14 are elevated within the tumor compartment. In the TME compartment, there were elevated levels of OX40L, CD163, and VISTA, indicating an influx of T cells and macrophages in the TME.

## 4. Discussion

In this randomized phase II neoadjuvant SBRT trial, 30 Gy in three fractions produced substantially greater transcriptional and proteomic evidence of immune activation than 21 Gy in one fraction. While the 21 Gy in one fraction produced upregulation of macrophages (when profiled by tumor and TME compartments), the 30 Gy in three fractions arm yielded many more differentially expressed genes and greater increases in gene signatures and spatially resolved protein markers associated with macrophages, dendritic cells, neutrophils, CD8 T cells, NK cells, and Treg cells. These findings support the hypothesis that preoperative three-fraction SBRT can prime multiple innate and adaptive immune pathways in early-stage HR+ BC that are traditionally immune “cold” [9,11].

Our results align with preclinical and clinical evidence that fractionation and dose selection influence radiation-driven immunomodulation. Preclinical models demonstrated that fractionated regimens more reliably generate immune-mediated antitumor effects and abscopal responses when combined with immunotherapy than single large fraction regimens [15,21]. Our findings demonstrate higher transcriptional and proteomic activation of immune cell pathways after three fractions, suggesting that this hypofractionated regimen may be preferable in the neoadjuvant setting when immune priming is the objective. This also supports earlier published work from this trial where PET/MRI imaging demonstrated greater inflammatory peritumoral response to 30 Gy in three fractions versus 21 Gy in one fraction [32]. These findings together support growing interest in revisiting the use of radiotherapy in breast cancer as an immunomodulatory tool rather than simply an ablative one.

The enrichment of antigen-presentation and adaptive immune pathways and upregulation of macrophage, dendritic cell, CD8 and NK cell signals, are mechanistically consistent with radiation-induced ‘in situ vaccination’. Radiation can enhance tumor antigen release, dendritic cell maturation and cross-presentation, and type I interferon signaling that supports CD8 T-cell recruitment and activation [8,18]. The concurrent rise in regulatory and immunoinhibitory markers (e.g., Treg signatures, VISTA, PD-L1 in some compartments) highlights the complex balance of stimulatory and suppressive changes induced by radiotherapy and suggests rational combinations with immunomodulators (e.g., anti-PD-1/PD-L1, anti-VISTA, Treg-targeting agents) may be required to shift the balance toward durable antitumor immunity [33,34]. These complex and seemingly conflicting findings with concurrent upregulation of immune activating and suppressing genes are consistent with published findings that describe the complex immunomodulatory effects of radiation [35]. While the genes upregulated by the -fractions of radiotherapy demonstrate gene activation (through CD8, dendritic cell and macrophage cell pathways), the downstream effects may be dampened by the chronic inflammatory and T cell exhaustion mechanisms (through anti-PD-L1 and Treg pathways of immunosuppression) [35]. These findings support the need for further work with checkpoint inhibitor treatment concurrent with preoperative radiation to effectively exploit immune priming while mitigating the immunomodulatory aspects of this complex tumor microenvironment.

Conventional histologic TIL counts did not show a consistent increase at the 14–20 day post-SBRT time point. This dissociation between early molecular/proteomic activation and overt changes in TILs is important: transcriptional and spatial proteomic changes likely represent early immune priming that precedes larger-scale lymphocyte influx or phenotypic differentiation detectable by routine histology. Prior clinical neoadjuvant RT studies with longer sampling intervals have sometimes demonstrated delayed cellular infiltration or radiologic tumor response months after treatment [36,37], so sampling timing is a critical variable when assessing immune endpoints. The prognostic value of TIL counts in patients treated with neoadjuvant radiotherapy requires further exploration.

For trials aiming to combine preoperative RT with checkpoint inhibitors or other immunotherapies in HR+ BC, our findings support using a three-fraction SBRT approach rather than a single ablative fraction to maximize immune priming. Fractionated preoperative SBRT is feasible and well tolerated and it may increase the likelihood of synergistic interactions with immune checkpoint inhibitors [8,20,38,39].

This study has several limitations. The sample size for molecular and proteomic sub-analyses was modest and the analysis window (14–20 days) may have missed slower evolving adaptive immune changes, as TIL expansion and functional T-cell maturation can continue for weeks to months after radiation [37]. However, given that the purpose of this study was to identify which treatment arm exhibited transcriptomic signals as early signs of immune upregulation, the time window between treatment and analysis was effective. Additionally, the trial population was restricted to older, postmenopausal patients with small, ER+ HER2− tumors, which limits extrapolation to other breast cancer subtypes (e.g., TNBC, HER2+) and limiting any notable changes in TIL counts given low TILs in this phenotype. Unfortunately, not every patient had their baseline diagnostic biopsy at our institution and we therefore did not obtain fresh frozen tumor samples from this subset of patients. This reduced the number of patients with tissue available for genomic analysis from 61 to 47. While this reduced sample size may have impacted statistical power for analysis, we were able to see a statistically significant upregulation in the three-fraction cohort. Finally, while spatial proteomics and gene expression provide rich signatures of immune activation, functional confirmation (e.g., antigen-specific T-cell responses, long-term clinical outcomes including recurrence rates) will be required to demonstrate clinically meaningful immunologic priming. Tumor cells are profiled simultaneously with both co-localized and distant stroma and immune cells, and therefore bulk expression profiling is an imperfect tool for analyzing tumor and microenvironmental changes across treatment. In particular, it is difficult to assign observed changes to specific geographic or phenotypic cell populations within the complex tumor ecosystem, where malignant tumor cells interact with fibroblasts, endothelial cells, and immune cells. Moreover, immune cells can be further divided into those that infiltrate the tumor core and those that are excluded. As of yet, how the tumor and immune microenvironments change during therapy remains poorly understood, necessitating multiplexed in situ profiling of longitudinal tissue samples. Future work should include larger, multi-site studies with extended post-RT sampling, integration of peripheral immune monitoring, and randomized evaluation of RT ± immunotherapy to determine whether the molecular signals we observed translate into improved recurrence-free survival.

## 5. Conclusions

In summary, neoadjuvant three-fraction SBRT (30 Gy in three fractions) induced greater molecular and proteomic immune upregulation in HR+ early-stage breast cancer than a single 21 Gy fraction, supporting fractionated preoperative SBRT as a pragmatic approach for immune priming and providing a rationale for three-fraction preoperative radiation regimens in trials combining RT with immunomodulatory therapies.

## Figures and Tables

**Table 1:** Patient and Tumor Characteristics by Treatment Arm.

	Participants with Tumor Available for Analysis			All Clinical Trial Participants	
Patient Demographics	n (%) 21 Gy/1	n (%) 30 Gy/3	*p*-Value	n (%) 21 Gy/1	n (%) 30 Gy/3
**Total Number of Patients**	**24**	**23**	**--**	**30**	**31**
Mean Age (Years; ± SD)	64.9 ± 8.17	64.7 ± 6.8	*p* = 0.9197	64.5 ± 7.47	65.3 ± 6.17
**Tumor Laterality**					
Left	12 (50)	14 (61)	*p* = 0.6485	18 (60)	19 (59)
Right	12 (50)	9 (39)		12 (40)	13 (41)
**Clinical Tumor Size**					
Mean Tumor Size (mm; ± SD)	11.38 ± 3.55	11.13 ± 3.75	*p* = 0.8192	10.3 ± 4.0	9.5 ± 4.7
**Pathological Tumor Size**					
Mean Tumor Size (mm; ± SD)	9.17 ± 4.33	8.57 ± 5.41	*p* = 0.6752	9.4 ± 5.1	8.7 ± 5.3
**Closet Surgical Margin**					
Mean Surgical Margin (mm; ± SD)	3.39 ± 2.44	4.09 ± 2.66	*p* = 0.3739	2.9 ± 2.6	3.9 ± 2.8
**Receptor Status**					
ER Positive	24 (100)	23 (100)	*p* = 0.1311	29 (100)	32 (100)
PR Positive	16 (67)	12 (52)	*p* = 0.6885	24 (82.8)	26 (81.3)
Nodal Status *					
cN0	N/A	N/A	--	4 (13.3)	7 (22.6)
N0	N/A	N/A	--	23 (76.7)	42 (67.7)
N1	N/A	N/A	--	3 (10)	2 (6.3)
N2	0	0	--	0	1 (3.1)
**Nottingham Grade**					
1	10 (42)	7 (30)	*p* = 0.7868	13 (46.4)	13 (43.3)
2	12 (50)	13 (56)		14 (50)	16 (53.3)
3	1 (4)	1 (4)		1 (3.6)	1 (3.3)
**Pathological Complete Response (pCR)**					
pCR	2 (8.3)	2 (8.7)	--	2 6.7)	3 (6.5)
**Chemotherapy Regimen**					
None	23 (96)	21 (91)	*p* = 0.3808	29 (96.8)	28 (90.3)
AC Weekly Paclitaxel	0 (0)	1 (4)		0	2 (6.5)
Docetaxel/Cyclophosphamide	1 (4)	0 (0)		1 (3.3)	0
3 weeks 12 doses Paclitaxel	0 (0)	1 (4)		0	1 (3.2)
**Endocrine Regimen**					
None	13 (54)	18 (78)	*p* = 0.27418	16 (53.3)	22 (80)
Letrozole	4 (17)	3 (13)		7 (23.3)	5 (16.1)
Arimidex	3 (12)	2 (9)		4 (13.3)	3 (9.7)
Tamoxifen	3 (12)	0 (0)		2 (6.7)	1 (3.2)
Letrozole then Tamoxifen	1 (4)	0 (0)		1 (3.3)	0
**Adjuvant Radiation**					
No	22 (92)	22 (96)	*p* = 1.000	28 (93.3)	30 (93.8)
Yes	1 (4)	1 (4)		2 (6.7)	2 (6.3)
**Days Between Dx and Sx**					
Mean Number Days (Days ± SD)	83.1 ± 11.4	85.2 ± 1.4	*p* = 0.5921	85.2 ± 13.9	87.3 ± 15.7
**Days between Rx and Sx**					
Mean Number of Days (Days ± SD)	19.13 ± 15	18.78 ± 2.39	*p* = 0.5497	18.8 ± 2.4	18.6 ± 3.8

n = number; Sx = surgery; Dx = diagnosis; SD = standard deviation; Rx = radiotherapy; AC = Adriamycin/cyclophosphamide; 21 Gy/1 = 21 Gy in one fraction; 30 Gy/3 = 30 Gy in three fractions (10 Gy per fraction). * Patient samples were deidentified when sent for gene expression profiling and therefore nodal status for those patients is not known. Also, sentinel node biopsy was not performed for cN0 participants based on clinical examination as per Choosing Wisely guidelines as determined by treating physician.

**Table 2:** Percentage of Stromal Tumor Infiltrating Lymphocytes (TILs) prior to and following neoadjuvant 21 Gy in one fraction versus 30 Gy in three fractions.

TIL %	Pre-Radiotherapy		Post-Radiotherapy *	
	21 Gy in 1 Fraction	30 Gy in 3 Fractions	21 Gy in 1 Fraction	30 Gy in 3 Fractions
0	8	7	2	2
1	4	6	4	5
5	4	2	6	4
10	2	1	4	4
20	1	1	1	1
50	1	1	1	0

* Two patients in each treatment arm had no tumor left in the lumpectomy specimen.

## Data Availability

All data used in this study was generated by study participants and no publicly available data was utilized.

## Supplementary Materials

The following supporting information can be downloaded at: https://www.mdpi.com/article/10.3390/cancers18121867/s1, Supplementary Table S1: Gene annotation data obtained from the NanoString product site was used to define gene signatures for immune cells. For each cell, the listed set of genes was assumed to be specific to its cell type. Supplementary Table S2: Immunostaining was performed using the list of antibodies, comprising an 18-plex core panel for immune cell profiling, supplemented by four additional modules: immuno-oncology drug targets (10-plex), immune activation status (8-plex), immune cell typing (7-plex), and pan-tumor (9-plex).
