# Supplementary material for: Determining Optimal Fractionation of Neoadjuvant Radiation in Low-Risk, Early-Stage Breast Cancer—Randomized SIGNAL Clinical Trial
Source: Cancers (Basel). 2026 Jun 8;18(12):1867. doi: 10.3390/cancers18121867 (PMC13296448; doi:10.3390/cancers18121867)
Supplement: Supplementary file 1 [file cancers-18-01867-s001.zip › cancers-4320641-supplementary.pdf]

Supplementary Table S1. Gene annotation data obtained from the NanoString product site was used to define gene signatures for immune cells. For each cell, the listed set of genes was assumed to be specific to its cell type.

Cell Type	***Genes***
B-cells	BLK, CD19, MS4A1, TNFRSF17
CD45	PTPRC
CD8 T cells	CD8A, CD8B
Cytotoxic cells	CTSW, GNLY, GZMA, GZMB, GZMH, KLRB1, KLRD1, KLRK1, PRF1
DC	CCL13, CD209, HSD11B1
Exhausted CD8	CD244, EOMES, LAG3
Macrophages	CD163, CD68, CD84
Mast cells	MS4A2, TPSAB1
Neutrophils	CSF3R, FCGR3A, S100A12
NK CD56dim cells	IL21R, KIR_Inhibiting_Subgroup_2, KIR3DL1, KIR3DL2
NK cells	NCR1, XCL2
T-cells	CD3D, CD3E, CD3G, CD6, SH2D1A
Th1 cells	TBX21
Treg	FOXP3

Supplementary Table S2. Immunostaining was performed using the list of antibodies, comprising an 18-plex core panel for immune cell profiling, supplemented by four additional modules: immuno-oncology drug targets (10-plex), immune activation status (8-plex), immune cell typing (7-plex), and pan-tumor (9-plex).

Target Group	***Proteins***
Antigen	HLA-DR, Beta-2-microglobulin, S100B, NY-ESO-1, PTEN, Her2, ER-alpha, MART1, PR
Antigen Presentation	HLA-DR, Beta-2-microglobulin
Apoptosis	Bcl-2
B cells	CD20
Background	Rb IgG, Ms IgG2a, Ms IgG1

CD8 T cells	CD8
Checkpoint	CTLA4, PD-L1, PD-L2, PD-1, Tim-3, VISTA, B7-H3, LAG3
CT Antigen	NY-ESO-1, MART1
Cytotoxicity	GZMB
DC	CD11c
Epithelial	PanCk, EpCAM
Fibroblasts	Fibronectin, FAP-alpha
Hematopoietic	CD34
Hormone Receptor	ER-alpha, PR
Housekeepers	Histone H3, GAPDH, S6
Interferon	STING
Macrophage	CD68, CD163, VISTA, ARG1
Melanoma	S100B, MART1
Memory	CD45RO
M2 Macrophage	CD68, CD163, ARG1
MHC2	HLA-DR
Monocyte	CD14
Myeloid	CD68, CD4, CD11c, CD40, CD80, CD14, CD66b, VISTA, ARG1, IDO1
Myeloid Activation	PD-L1, CD40, CD80, VISTA, OX40L, ARG1
Myeloid Suppression	ARG1, IDO1
Naive and Memory	CD127
Neutrophil	CD66b
NK cells	CD56
Oncogene	Her2
Proliferation	Ki-67
Stroma	Fibronectin, SMA, FAP-alpha
T cell Activation	CTLA4, GZMB, PD-1, CD25, ICOS, CD27, CD44, GITR, OX40L, 4-1BB, Tim-3, LAG3
T cells	CTLA4, CD4, CD3, CD8, PD-1, CD127, CD25, CD27, FOXP3, CD45RO, GITR, Tim-3, 4-1BB, LAG3
Th cells	CTLA4, CD4, FOXP3
Total Immune	CD45
Tregs	CD25, FOXP3
Tumor	PanCk, Beta-2-microglobulin, S100B, Bcl-2, EpCAM, MART1
Tumor Suppressor	PTEN
